# Supplementary material for: NOX2-TRPM2 coupling promotes Zn2+ inhibition of complex III to exacerbate ROS production in a cellular model of Parkinson’s disease
Source: Sci Rep. 2024 Aug 8;14:18431. doi: 10.1038/s41598-024-66630-9 (PMC11310326; doi:10.1038/s41598-024-66630-9)
Supplement: Supplementary file 1 — Supplementary Figures. [file 41598_2024_66630_MOESM1_ESM.docx]

**Supplementary Figure 1**


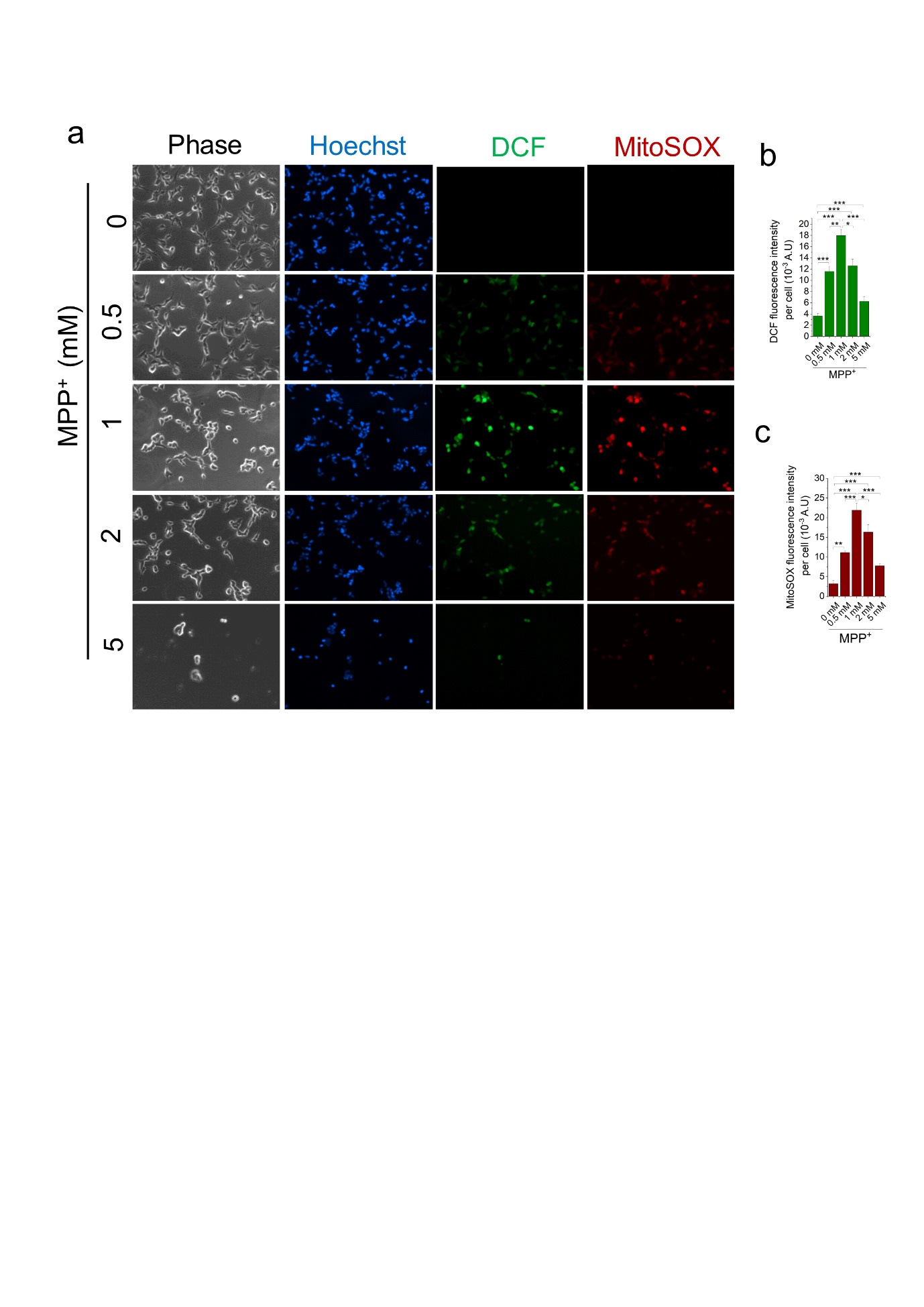
**Dose dependent effect of MPP^+^ on total intracellular and mitochondrial ROS generation in SH-SY5Y cells.**

SH-SY5Y cells treated with the indicated concentrations of MPP^+^ for 24 h were stained for nuclei (Hoechst 33342), and for total (DCF) and mitochondrial (MitoSOX) ROS. **(a)** Representative phase and fluorescent images of stained cells. **(b-c)** the corresponding mean ± SEM of fluorescence intensity of DCF (b) and MitoSOX (c) from three independent experiments performed as in (a). ** *p* ˂ 0.01; *** *p* ˂ 0.001 from One-way Anova with post-hoc Tukey Test.

**Supplemental Figure 2:**

**Raw Western blot data relating to Figure 4A**


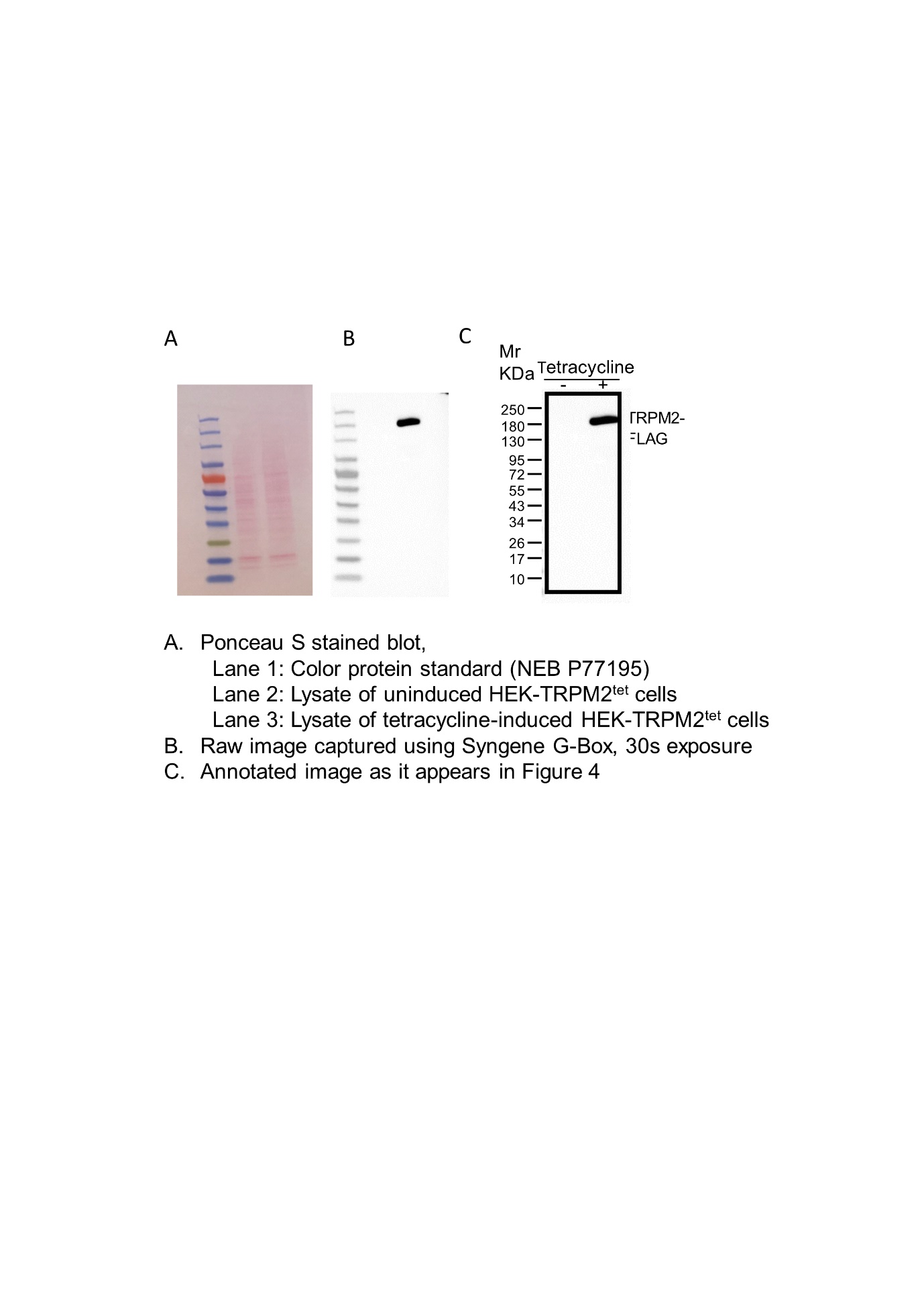


Raw data of the Ponceau S-stained nitrocellulose blot, image of the ECL stained blot and the final cropped figure as presented in main Figure 5.

**Supplemental Figure 3**

**MPP^+^-induced TRPM2-mediated Ca^2+^ entry causes a rise in mitochondrial free Zn^2+^ in SH-SY5Y cells**


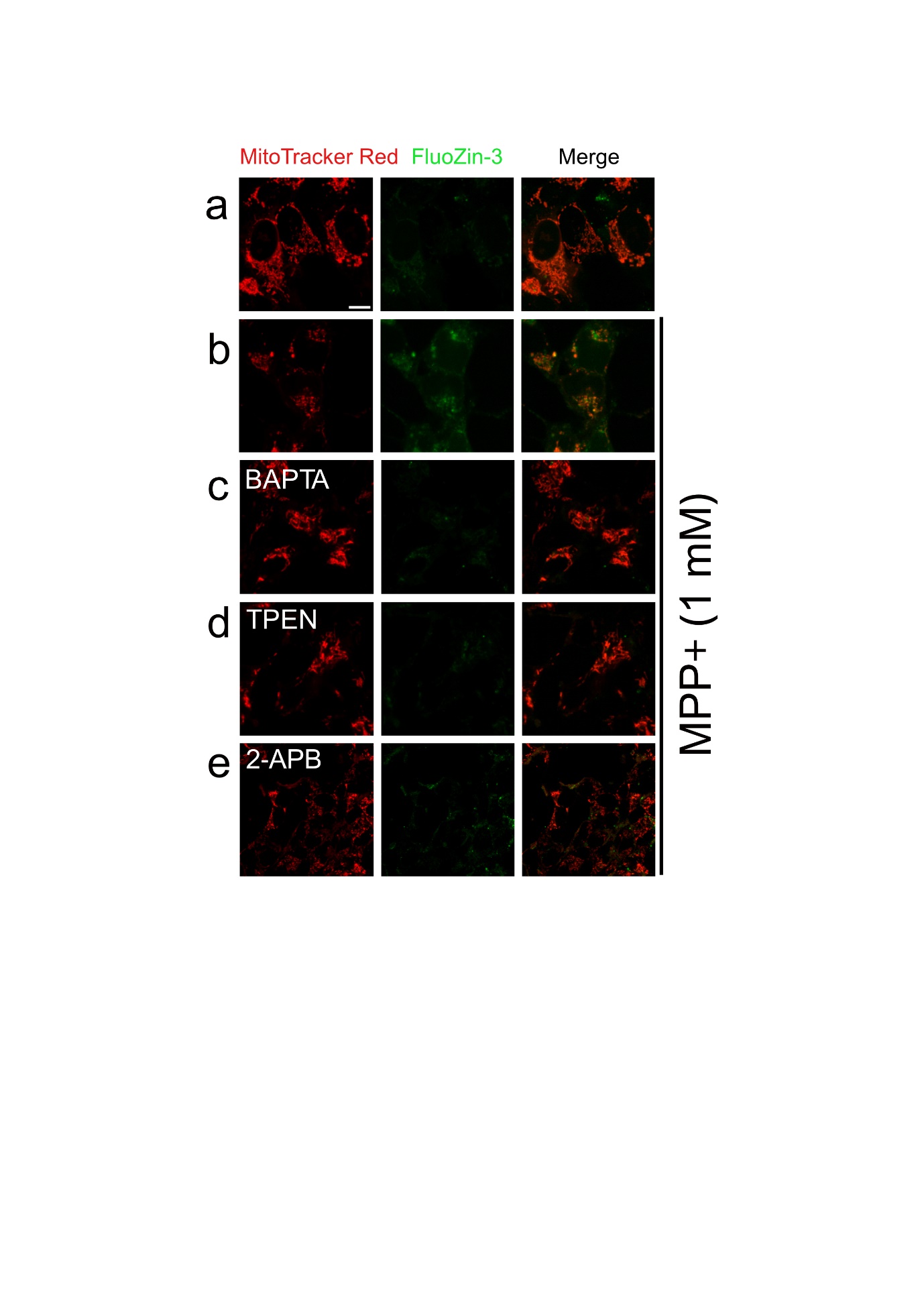


**(a-b).** MPP^+^ treatment increases free Zn^2+^ levels in mitochondria in SH-SY5Y cells. Cells were either untreated (A) or treated with 1 mM MPP^+^ (B) before staining for Zn^2+^ using 1 µM FluoZin-3 and mitochondria with MitoTracker Red (50 nM) as described {Abuarab, 2017 #654}. Cells were imaged using an Zeiss LSM800 confocal microscope. MPP^+^ treatment caused a marked increase in the fragmentation of the mitochondrial network with the broken mitochondria displaying increased Zn^2+^ fluorescence (yellow/orange puncta in merged images in b). By contrast control cells (a) showed intact network; no yellow puncta are evident in these cells which is consistent with the evidence that healthy cells have low levels of free Zn^2+^ in mitochondria. These findings are in agreement with the findings reported previously with nonneuronal cells {Abuarab, 2017 #654 {Li, 2017 #652}}.

**(c-d).** MPP^+^ induced mitochondrial Zn^2+^ rise is Ca^2+^ dependent. Pretreatment of cells with the Ca^2+^ chelator, BAPTA-AM (5 µM) prevented mitochondrial Zn^2+^ elevation (c) as effectively as the Zn^2+^ chelator, TPEN (0.5 µM) (d), indicating the role of Ca^2+^ in MPP^+^ induced mitochondrial Zn^2+^ elevation.

**(e)**. MPP^+^ induced mitochondrial Zn^2+^ rise is TRPM2-dependent. Pretreatment of cells with the pharmacological inhibitor of the TRPM2 channel, 2-APB prevented mitochondrial Zn^2+^ elevation.

**Supplemental Figure 4**

**Validating the role of TRPM2 channels in oxidative stress induced mitochondrial Zn^2+^ rise**.

**
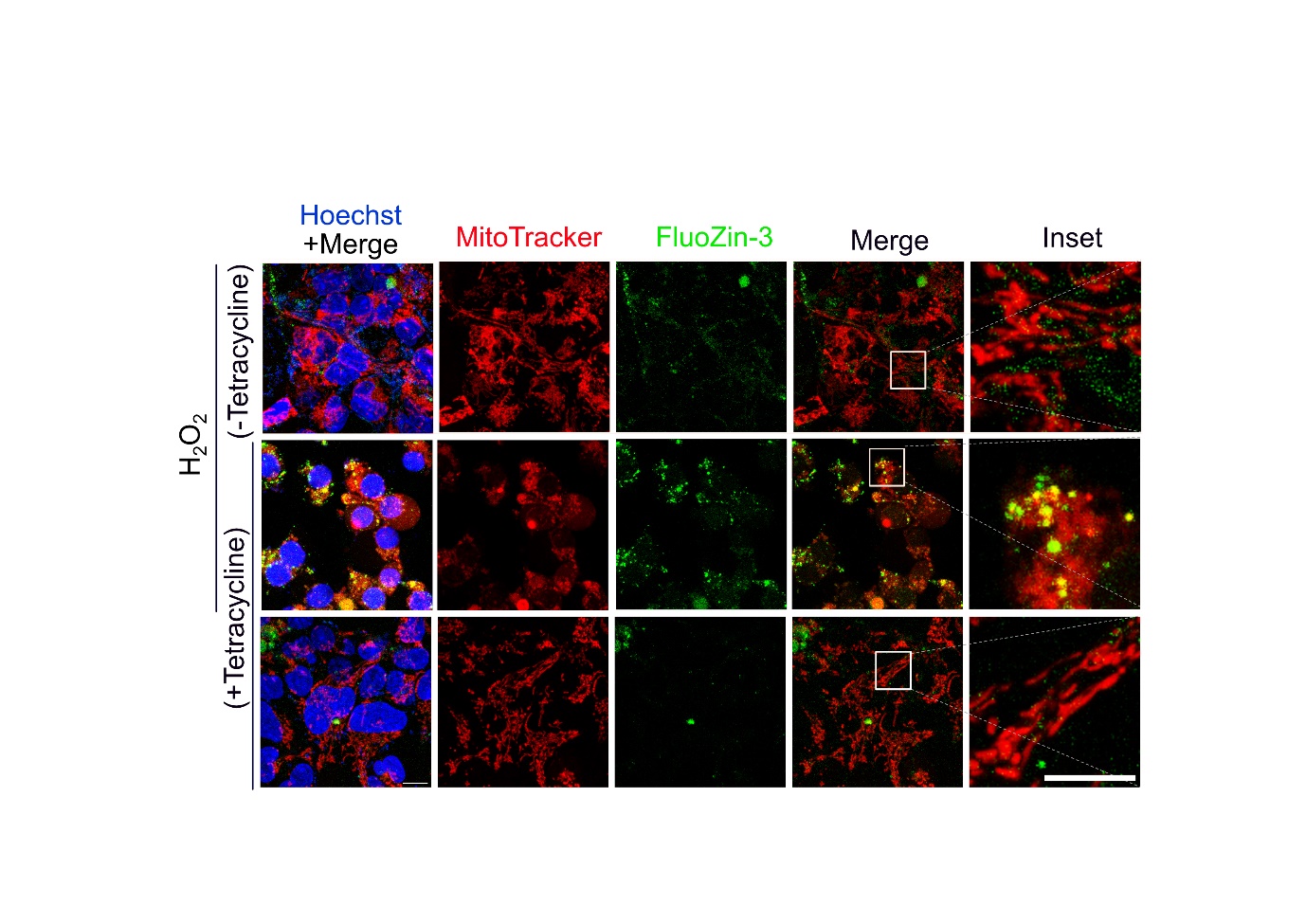
**

TRPM2 expression in HEK-293-TRPM2^tet^ cells was either uninduced (top row) or induced with tetracycline (bottom two rows). Cells preloaded with FluoZin-3 were treated with 100 µM H_2_O_2_ (treated rows indicated with a vertical bar). H_2_O_2_ action was terminated with 2 mM dithiothreitol. All cells were co-stained with MitoTracker Red and Hoechst. Images were acquired using the Zeiss LSM800 confocal microscope. The results show that in the absence of tetracycline induction of TRPM2 expression (top row), H_2_O_2_ induced oxidative stress failed to cause detectable increase in mitochondrial Zn^2+^. Upon tetracycline induction of TRPM2 expression (middle row), cells displayed a marked increase in mitochondrial fragmentation along with an increase in mitochondrial Zn^2+^ levels (yellow puncta in merged images, expanded in inset). In the absence of H_2_O_2_ treatment, no such changes were observed in TRPM2 expressing cells (bottom row). These findings underpin the role of TRPM2 channels in oxidative stress induced rise in mitochondrial Zn^2+^. Scale bar 10µm, 5 µm in expanded image.
